# A review for discovering bioactive minor saponins and biotransformative metabolites in *Panax quinquefolius* L.

**DOI:** 10.3389/fphar.2022.972813

**Published:** 2022-08-01

**Authors:** Zhiyou Yang, Jiahang Deng, Mingxin Liu, Chuantong He, Xinyue Feng, Shucheng Liu, Shuai Wei

**Affiliations:** ^1^ Guangdong Provincial Key Laboratory of Aquatic Product Processing and Safety, Guangdong Province Engineering Laboratory for Marine Biological Products, Key Laboratory of Advanced Processing of Aquatic Product of Guangdong Higher Education Institution, Guangdong Provincial Engineering Technology Research Center of Seafood, College of Food Science and Technology, Guangdong Ocean University, Zhanjiang, China; ^2^ Collaborative Innovation Centre of Seafood Deep Processing, Dalian Polytechnic University, Dalian, China; ^3^ College of Electrical and Information Engineering, Guangdong Ocean University, Zhanjiang, China

**Keywords:** *Panax quinquefolius*, minor ginsenosides, metabolites, structural diversity, pharmacological effects

## Abstract

*Panax quinquefolius* L. has attracted extensive attention worldwide because of its prominent pharmacological properties on type 2 diabetes, cancers, central nervous system, and cardiovascular diseases. Ginsenosides are active phytochemicals of *P. quinquefolius*, which can be classified as propanaxdiol (PPD)-type, propanaxtriol (PPT)-type, oleanane-type, and ocotillol-type oligo-glycosides depending on the skeleton of aglycone. Recently, advanced analytical and isolated methods including ultra-performance liquid chromatography tandem with mass detector, preparative high-performance liquid chromatography, and high speed counter-current chromatography have been used to isolate and identify minor components in *P. quinquefolius*, which accelerates the clarification of the material basis. However, the poor bioavailability and undetermined bio-metabolism of most saponins have greatly hindered both the development of medicines and the identification of their real active constituents. Thus, it is essential to consider the bio-metabolism of constituents before and after absorption. In this review, we described the structures of minor ginsenosides in *P. quinquefolius*, including naturally occurring protype compounds and their *in vivo* metabolites. The preclinical and clinical pharmacological studies of the ginsenosides in the past few years were also summarized. The review will promote the reacquaint of minor saponins on the growing appreciation of their biological role in *P. quinquefolius*.

## Introduction

Ginseng root has historically been used as medicine food homology plant for thousand years in oriental countries. It occupies a prominent position in the list of best-selling natural medicines worldwide (Qi et al., 2011). *Panax ginseng* C.A. Meyer (known as Asian or Korean ginseng), *P. quinquefolius* (known as American ginseng), and *P. notoginseng* (Burkill) F.H. Chen (known as Sanchi ginseng) are three reputable folk medicine around the world. *P. quinquefolius* is one of the top 10 selling natural health products in the United States. Despite its high chemical similarity with Asian ginseng, *P. quinquefolius* instead exhibits heat-clearing and refreshing functions as a tonic medicinal plant (Yang et al., 2014). Modern pharmacological studies indicated *P. quinquefolius* exert a wide range of biological activities, such as hypoglycemic, cardiovascular protective, anti-diabetic, anti-tumor, anti-inflammatory, anti-obesity, anti-aging, and antimicrobial effects (Assinewe et al., 2003; Szczuka et al., 2019).

It is well documented that the triterpenoid saponins, called ginseng saponins or ginsenosides, are the major active compounds in *P. quinquefolius* (Yuan et al., 2010). The ginsenoside profile varies in this herb due to the cultivation in different areas in terms of total ginsenosides, the ratio of protopanaxadiol (PPD) to protopanaxatriol (PPT), and other marker ginsenosides. The type and contents of ginsenosides are also different in the root, stem/leaves, flower bud, and fruits. Thus, a wide spectrum of advanced analytical methods including ultra-performance liquid chromatography tandem with mass detector, preparative high-performance liquid chromatography, and high-speed counter-current chromatography have been used to isolate and identify minor components in *P. quinquefolius*, which accelerates the clarification of its material basis.

Rb1, Rb2, Rc, Re, and Rg1 are considered as major ginsenosides with high contents in *P. quinquefolius*. The multitude of sugar moieties in major ginsenosides affects their bioavailability after oral intake, as well as the biological activities. The bioactive ginsenosides *in vitro* do not always represent the real active form *in vivo*, due to the bio-metabolism of constituents by trillions of gut microbiota in the gastrointestinal tract and enzymes in blood and tissues after absorption. To link the health benefits of major ginsenosides to their effects, it is warranted to determine the profiles of *P. quinquefolius* and its minor metabolites.

In this review, the structural diversities of ginsenosides in different parts of *P. quinquefolius* are described, especially naturally occurring minor ginsenosides and those resulting from biotransformation. Preclinical and clinical studies of *P. quinquefolius* and ginsenosides are also delineated. Finally, special attention is paid to future research trends for *P. quinquefolius*, and targets identification of bioactive ginsenosides and their underlying mechanism exploration are discussed and prospected.

## *P*. *quinquefolius*: Geographical distribution and application

**FIGURE 1:**
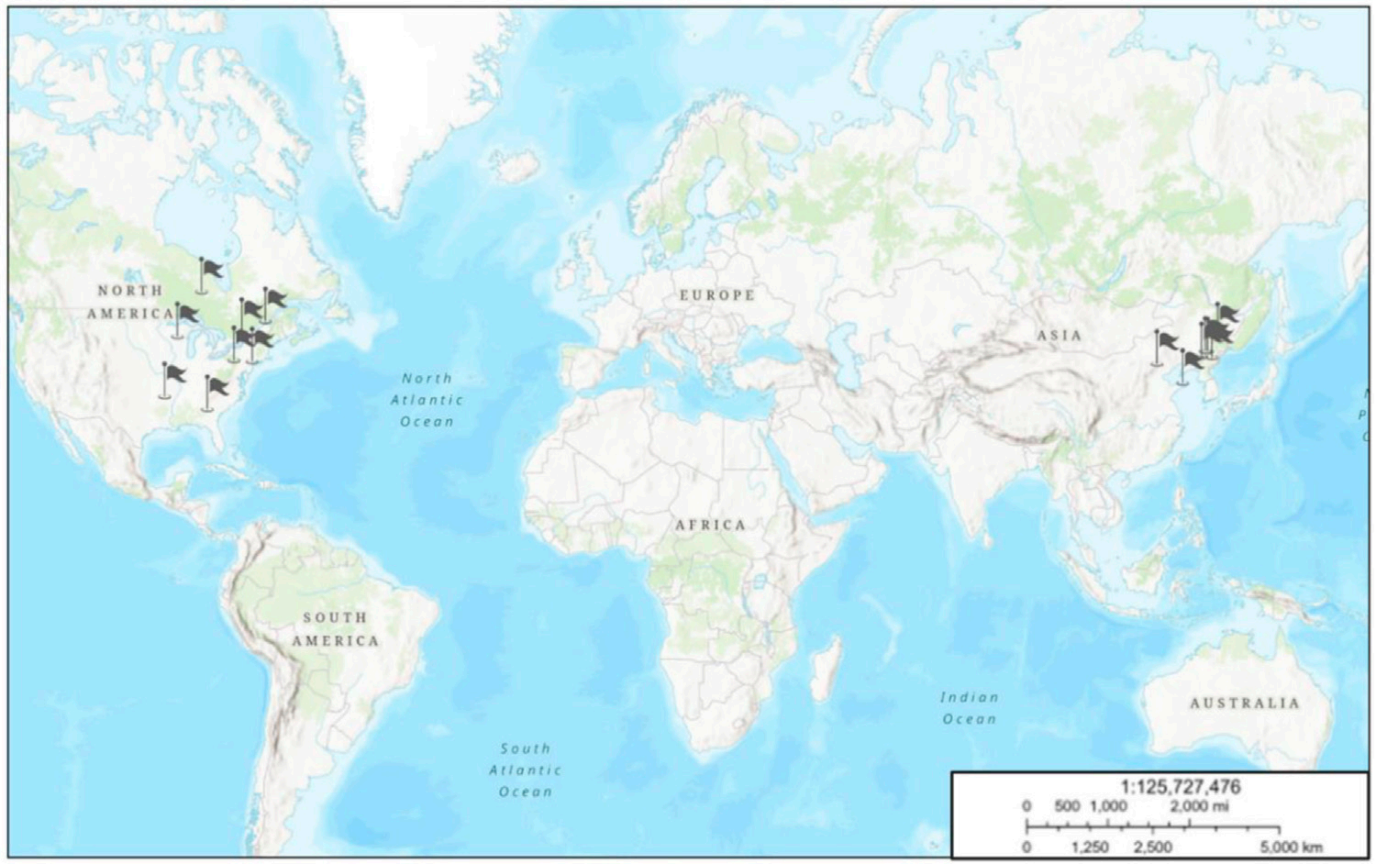
Geographical distribution of *P. quinquefolius* based on GMPGIS. The map was plotted using online ArcGIS (ESRI, Redland, CA, United States. URL: http://www.learngis2.maps.arcgis.com/). Flags showing cultivated or wild resources of *P. quinquefolius*.

*P. quinquefolius* was first found in 1716 by father Joseph-François Lafitau, a Jesuit priest in Canada. He stumbled across *P. quinquefolius* growing in the woods near Montreal. It is distributed native to the temperate forest regions of North America, from 67° to 95°W longitude and 30° to 48°N latitude, including North of Quebec and Ontario and South of Mississippi, Arkansas, and Georgia. Wild ginseng is still harvested from areas in Wisconsin, Pennsylvania, and New York State. *P. quinquefolius* was first introduced to China in 1975, and the major producing areas are Heilongjiang, Jilin, Liaoning, Hebei, Shandong, and Shanxi Provinces (Figure 1) (Shen et al., 2019).

*P. quinquefolius* can be cultivated in large number of countries except for the abovementioned places. Based on the environmental variables over 30 years from 1970 to 2000, and 226 global distribution areas of *P. quinquefolius*, the maximum entropy model (MaxEnt) was used to predict the global ecological suitable areas for *P. quinquefolius*. The potential ecological suitable places of *P. quinquefolius* were primarily in Changbai Mountain in China and Appalachian Mountain in America, in the range of 35°N–50°N, 110°E–145° and E35°N–50°N, 60°W–120°W, respectively, including Canada, the United States, China, North and South Korea, Russia and Japan. Japan and South Korea were the potential producing regions (Zhang et al., 2018).

*P. quinquefolius* has been used by native Americans for a long history. It was used in Cherokee medicine for coughing, shortness of breath, headaches, digestive upset, fatigue, convulsions, female reproductive problems, and general weakness. An assortment of products containing *P. quinquefolius* are currently available on the market, including capsule, tablet, powder, and tea. The roots are implemented in drugs, cosmetic and skin care, food and feed additives. In United States, *P. quinquefolius* extracts are used in candies and drinks, while in China, they are used in alcoholic beverages (Szczuka et al., 2019).

## Structural diversity of ginsenosides in *P. quinquefolius*

Ginsenosides, which share a unique dammarane type triterpenoid saponin structure (Fuzzati, 2004), are the major characteristic constituents of *P. quinquefolius*. More than 100 ginsenosides have been identified in *P. quinquefolius*, including naturally occurring compounds and those resulting from steaming and biotransformation (Yuan et al., 2010). The contents and types of ginsenosides vary from the roots, leaves, stems, flower buds and fruits of *P. quinquefolius* (Table 1). A comprehensive study was conducted to compare the components among different parts of *P. quinquefolius* and found that the root contains much more abundant Rb1, Ro, and mRb1 isomer, compared with the other parts. The stem leaf and flower bud show similar saponin composition, with richer m-Rb2, Rb3, and p-F11, than the root (Wang et al., 2019). Differences were found in sugar moieties, numbers, and sugar attachment at positions C-3, C-6, or C-20 and they provided diversity in ginsenoside structures (Qi et al., 2011). The carbonylation at C-3, dehydrogenation at C-5, 6 and changeable C-20 side-chain, and stereoisomerism further enrich the structural diversity of ginsenosides.

**TABLE 1:** The natural occurring ginsenosides in different parts of *P. quinquefolius*.

No	Name	Type	Medicinal parts				Identification methods	References
			Root	Stem/leaves	Flower buds	Fruits		
1	Rb1	PPD	√	√			NMR	Chen et al. (1981)
2	Rb2	PPD	√	√			NMR	Chen et al. (1981)
3	Rb3	PPD	√	√			NMR	Chen et al. (1981)
4	Rc	PPD	√	√			HPLC	Li et al. (1996)
5	Rd	PPD	√	√			NMR	Chen et al. (1981)
6	Q-I	PPD	√				NMR	Yoshikawa et al. (1998)
7	Q-II	PPD	√				NMR	Yoshikawa et al. (1998)
8	Q-III	PPD	√				NMR	Yoshikawa et al. (1998)
9	Q-V	PPD	√				NMR	Yoshikawa et al. (1998)
10	Malonyl-G-Rb1	PPD	√		√		NMR	Yoshikawa et al. (1998)
11	Pseudo-G-Rc1	PPD	√				NMR	Yoshikawa et al. (1998)
12	G-F2	PPD	√				NMR	Yoshikawa et al. (1998)
13	Gypenoside XVII	PPD	√				NMR	Yoshikawa et al. (1998)
14	Malonyl-G-Rb2	PPD	√		√		LC/MS/MS, NMR	Wang et al. (1999)
15	Malonyl-G-Rc	PPD	√		√		LC/MS/MS, NMR	Wang et al. (1999)
16	20(S)-G-Rh2	PPD		√			LC-MS/MS	Popovich and Kitts, (2004)
17	Rs1	PPD			√		NMR	Nakamura et al. (2007)
18	Pseudo-G-F8	PPD			√		NMR	Nakamura et al. (2007)
19	Q-L10	PPD		√			NMR	Chen et al. (2009)
20	Q-L14	PPD		√			NMR	Chen et al. (2009)
21	Q-L16	PPD		√			NMR	Chen et al. (2009)
22	20(*S*)-G-Rg3	PPD	√				NMR	Qi et al. (2011)
23	G-F8	PPD	√				NMR	Qi et al. (2011)
24	Malonyl-G-Rd	PPD			√		NMR	Wang et al. (2015b)
25	20(R)-G-Rg3	PPD	√				NMR	Qi et al. (2011)
26	20(R)-G-Rh2	PPD	√				NMR	Qi et al. (2011)
27	20(S)-PPD	PPD	√				NMR	Qi et al. (2011)
28	20(R)-PPD	PPD	√				NMR	Qi et al. (2011)
29	Q-IV	Modified PPD	√				NMR	Yoshikawa et al. (1998)
30	Notoginsenoside G	Modified PPD	√				NMR	Yoshikawa et al. (1998)
31	Notoginsenoside C	Modified PPD	√				NMR	Yoshikawa et al. (1998)
32	floralquinquenoside D	Modified PPD			√		NMR	Nakamura et al. (2007)
33	ginsenoside I	Modified PPD			√		NMR	Nakamura et al. (2007)
34	Notoginsenoside E	Modified PPD			√		NMR	Nakamura et al. (2007)
35	Notoginsenoside K	Modified PPD	√				NMR	Yoshikawa et al. (1998)
36	quinquenoside L3	Modified PPD		√			NMR	Wang et al. (1998)
37	Notoginsenoside A	Modified PPD	√				NMR	Yoshikawa et al. (1998)
38	quinquenoside L2	Modified PPD		√			NMR	Wang et al. (2001)
39	quinquenoside L1	Modified PPD		√			NMR	Wang et al. (2001)
40	Rg1	PPT	√	√			NMR	Chen et al. (1981)
41	Re	PPT	√	√			NMR	Chen et al. (1981)
42	Rf	PPT	√				NMR	Yoshikawa et al. (1998)
43	Rg2	PPT	√				NMR	Yoshikawa et al. (1998)
44	Rh1	PPT	√				NMR	Dou et al. (2006)
45	F1	PPT	√				NMR	Dou et al. (2006)
46	F3	PPT			√		NMR	Nakamura et al. (2007)
47	Q-L17	PPT		√			NMR	Li et al. (2009)
48	Q-F6	PPT				√	NMR	Lu et al. (2012)
49	6’-O-Ac-G-Rg1	PPT	√				NMR	Qi et al. (2011)
50	20(S)-Ac-G-Rg2	PPT	√				NMR	Qi et al. (2011)
51	20(R)-Ac-G-Rg2	PPT	√				NMR	Qi et al. (2011)
52	F-E	PPT	√				NMR	Qi et al. (2011)
53	Malonyl-G-Re	PPT			√		NMR	Wang et al. (2015c)
54	Rg8	Modified PPT	√				NMR	Dou et al. (2006)
55	F4	Modified PPT	√				NMR	Dou et al. (2006)
56	floralquinquenoside E	PPT			√		NMR	Nakamura et al. (2007)
57	floralquinquenoside A	Modified PPT			√		NMR	Nakamura et al. (2007)
58	floralquinquenoside B	Modified PPT			√		NMR	Nakamura et al. (2007)
59	floralquinquenoside C	Modified PPT			√		NMR	Nakamura et al. (2007)
60	quinquenoside L9	Modified PPT			√		NMR	Nakamura et al. (2007)
61	24(R)-pseudo-G-F11	Ocotillol		√			NMR	Chen et al. (1981)
62	24(S)-pseudo-G-F11	Ocotillol			√		NMR	Nakamura et al. (2007)
63	pseudo-RT5	Ocotillol			√		NMR	Nakamura et al. (2007)
64	24(R)-vina-G-R1	Ocotillol			√		NMR	Nakamura et al. (2007)
65	12-one-pseudo-G-F11	Ocotillol		√			NMR	Qi et al. (2020)
66	Ocotillol	Ocotillol		√			NMR	Han et al. (2014)
67	3α-ocotillol	Ocotillol		√			NMR	Han et al. (2014)
68	pseudo-ginsenoside RT6	Modified Ocotillol		√			NMR	Liu et al. (2013)
69	pseudoginsengenin R1	Modified Ocotillol		√			NMR	Liu et al. (2013)
70	Chikusetsusaponin IVa	Oleanane	√				NMR	Yoshikawa et al. (1998)
71	G-Ro	Oleanane	√				NMR	Qi et al. (2011)
72	ginsenoside 1a	Modified type			√		NMR	Nakamura et al. (2007)
73	quinquefoloside-Ld	Modified type		√			NMR	Xiang et al. (2013)
74	quinquefoloside-Le	Modified type		√			NMR	Xiang et al. (2013)
75	dammar-20(S), 25(S)-epoxy-3β, 12β, 26-triol	Modified type	√				NMR	Han et al. (2016)

**FIGURE 2:**
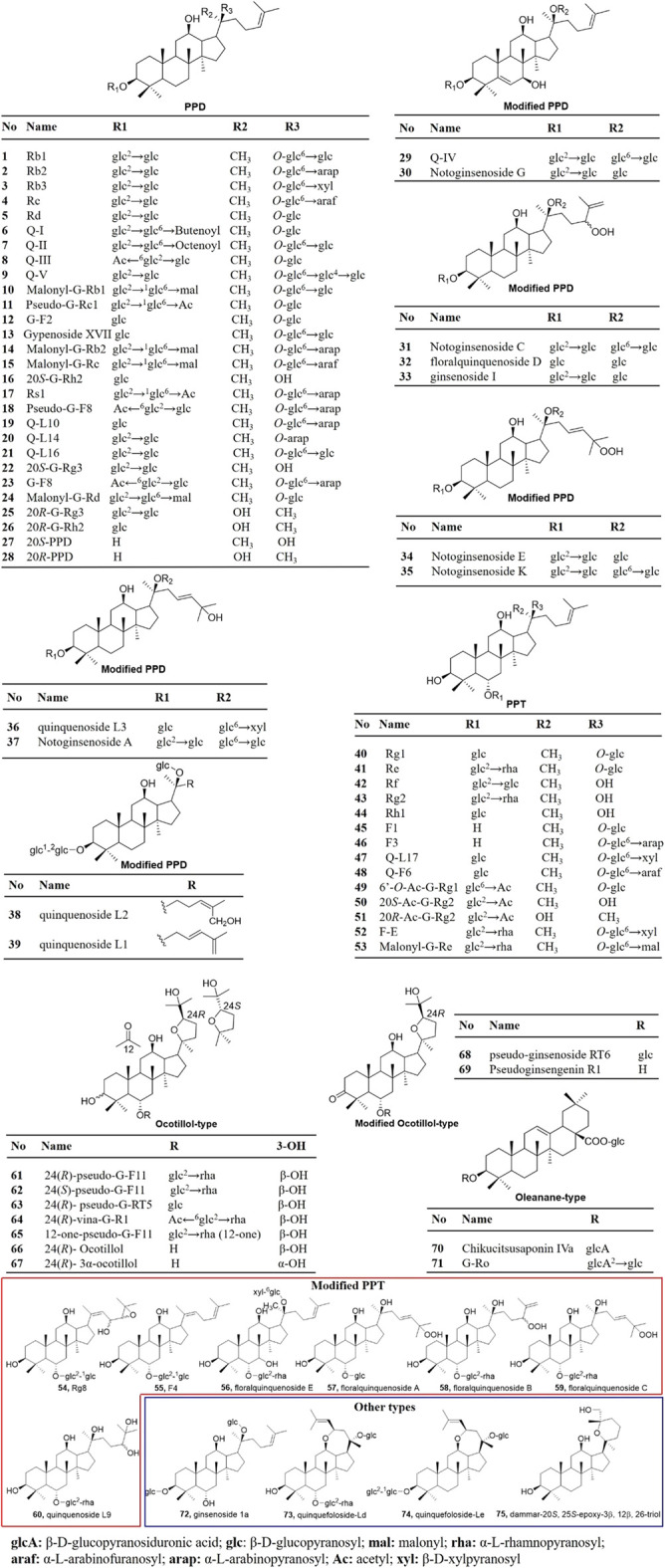
Ginsenosides characterized from *P. quinquefolius*. PPD, Protopanaxadiol; PPT, protopanaxatriol; G, ginsenoside; Q, quinquenoside.

As summarized in Figure 2, ginsenosides in *P. quinquefolius* are generally classified into four groups, consisting of protopanaxadiol-type (PPD), protopanaxatriol-type (PPT), ocotillol-type, and oleanolic acid-type. PPD and PPT are the major groups of ginsenosides and are usually found in neutral forms. In the PPD-type, sugar residues are attached to β-OH at C-3 and/or C-20. Natural occurring PPD compounds include compounds 1–28. Compounds 29–30 with modified PPD structure were characterized by a double bond between C-5 and C-6 and a hydroxyl group in C-7 was isolated from the roots of *P. quinquefolius* (Yoshikawa et al., 1998). Compounds 31–39 were clarified as modified PPD structures with variable C-20 side-chains. In the PPT group, sugar moieties are attached to the α-OH at C-6 and/or β-OH at C-20. PPT constituents include compounds 40–60. PPD and PPT type ginsenosides constitute the main saponins in *P. quinquefolius*, and reports have shown that Rb1, Rb2, Rc, Rg1, Re, and Rd account for 90% of the total saponins (Wang et al., 2015b). Minor ginsenosides isolated from *P. quinquefolius* include ocotillol-type (compounds 61–69), oleanane-type (compounds 70–71), and dammarane saponins with a modified aglycone skeleton (compounds 29–39 and 54–60). A variety of minor ginsenosides have been isolated and the structures were elucidated via MS/MS, and NMR analysis. For example, in 1998, Yoshikawa et al. identified 5 dammarane-type triterpene oligoglycosides named quinquenosides I–V from the root of *P. quinquefolius*, along with notoginsenoside A, C, G, K, malonyl G-Rb1, pseudo-G-Rc1, gypenoside XVII, and chikusetsusaponin Iva (Yoshikawa et al., 1998). Three new dammarane-type saponins named quinquenosides L1–3 were isolated from the leaves and stems of *P. quinquefolius* collected in Canada (Wang et al., 1998; Wang et al., 2001). By using LC/MS/MS, the ginsenosides malonyl G-Rb2 and malonyl G-Rc were characterized in the root of *P. quinquefolius* (Wang et al., 1999). In 2004, a new dammarane-type triterpenoid saponin, ginsenoside Rg8, was isolated from the roots of *P. quinquefolius*, along with (20E)-ginsenoside F4, Rh1, and F1 (Dou et al., 2006). In 2007, from the flower buds of *P. quinquefolius*, 5 new dammarane-type triterpene glycosides, floralquinquenosides A, B, C, D, and E, along with 18 known ginsenosides were isolated and identified by NMR analysis (Nakamura et al., 2007). Four new triterpenoid saponin quinquenoside L10, 14, 16, and 17 were isolated from the leaves and stems of *P. quinquefolius* in 2009 (Chen et al., 2009; Li et al., 2009). Quinquenoside F6 was isolated from the fruits of *P. quinquefolius* (Lu et al., 2012). Two new dammarane-type saponins quinquefoloside-Ld and Le with a novel heptatomic ring between C-12 and C-17 from leaves of *P. quinquefolius* were elucidated (Xiang et al., 2013). Two new ocotillol-type compounds were isolated from the leaves and stems of *P. quinquefolium* L. and identified as pseudo-ginsenoside RT6 and pseudoginsengenin R1 (Liu et al., 2013). A new ocotillol-type ginsenoside, namely 12-one-pseudoginsenoside F_11_ (12-one-Pseudo-G-F_11_), was isolated from stems and leaves of *P. quinquefolium* (Qi et al., 2020).

## The biotransformation of major ginsenosides into minor ginsenosides

The ginsenosides Rb1, Rb2, Rc, Re, and Rg1 are usually characterized as major ginsenosides, and account for more than 80% of total ginsenosides (Yu et al., 2017). The bioactive ginsenosides have been widely utilized in medical and chemical fields, which created a demand for their availability. However, the large size and poor cell membrane permeability of major ginsenosides restricted their absorption and bioavailability in human body after oral administration (Liu et al., 2010a). Therefore, the production of rare or minor smaller ginsenosides by transformation is urgently requisite. On the one hand, *in vitro* or *in vivo* biotransformation of major ginsenosides can generate an assortment of novel structural ginsenosides, resulted from the reduction of sugar moieties, substituent groups alteration and aglycone backbone changes. The small molecular ginsenosides are easily absorbed in the gastrointestinal tract after oral administration due to the deglucosylation (Ryu et al., 2017). In addition, a multitude of evidence showed that biotransformation of major ginsenosides to minor ginsenosides result in improved pharmacological activities (Quan et al., 2015; Ryu et al., 2017).

The deglycosylation of sugar moieties is mainly occurred in the transformation of major ginsenosides. Thermal and mild acid hydrolysis treatments show inefficient and low selective decomposition, while biotransformation including microbial enzymatic transformation, microbial transformation, and *in vivo* transformation, exerts high selectivity, lower by-products, and high targets yields. Thus, research on the biotransformation of major ginsenosides for increasing the bioavailability and pharmacological activities by structural modification of ginsenosides attracts more attention. The types, pathways, conditions, and yields of biotransformation were shown in Table 2.

**TABLE 2:** Biotransformation of major ginsenosides into rare ginsenosides.

Transformation pathways	Enzymes	Biotransformation conditions	Yield	Ref.
**Enzymatic transformation**				
Rb1→Rd→20(S)-Rg3	*M*. *esteraromaticum* (β-glucosidase bgp1)	pH 7.0, 37°C, 6 h	74.3%	Quan et al. (2012d)
Rb1→Rd→Compound K	*M*. *esteraromaticum* (β-glucosidase bgp3)	pH 7.0, 40°C, 1 h	77%	Quan et al. (2012b)
Rb1→Compound K	*L*. *mesenteroides* DC102 (Crude glycosidase)	pH 6–8, 30°C, 72 h	99%	Quan et al. (2011)
Rb1→Rd	*A*. *niger* (β-glucosidase immobilized with amino-based silica)	pH 5.5, 45°C, 1 h	3.30-fold	Wu et al. (2021)
Rb1, Rb2, Rc, Rd→Ginsenoside F2	*Sphingomonas* sp. 2F2 (β-glucosidase bglSp)	pH 5.0, 37°C	—	Wang et al. (2011)
Rb2→Compound Y→Compound K	*M*. *esteraromaticum* (β-glycosidase)	pH 7.0, 40°C	—	Quan et al. (2012a)
Rb2→Rd→Compound K, Rb2→C-O→Compound K	*A*. *mellea* mycelium (β-glucosidase)	pH 4–4.5, 45–60°C, 72–96 h	—	Kim et al. (2018)
Rb2→Rd	α-L-Arabinopyranosidase	pH 7.0, 40°C, 1 h	—	Kim et al. (2020)
Rc→Rd	*T. thermarum* DSM5069 (a-L-arabinofuranosidase)	pH 5.0, 95°C	99.4%	Xie et al. (2016)
Re→Rg2, Rg1→Rh1	β-glucosidase (Bgp1)	pH 7.0, 37°C	100%, 78%	Quan et al. (2012c)
Rf→Rh1	*A*. *niger* (β-glucosidase (Bgl1))	pH 7.5, 37°C	—	Ruan et al. (2009)
Rf→Protopanaxatriol	*A*. *niger* (β-glucosidase)	pH 5.0, 55°C	90.4%	Liu et al. (2010a)
Rg1→Ginsenoside F1	*S*. *keddieii* (glycosidase bglSk)	pH 8.0, 25°C	100%	Kim et al. (2012)
**Microbial Transformation**				
Rb1→Rd	*B*. *pyrrocinia* GP16, *Bacillus megaterium* GP27, *Sphingomonas echinoides* GP50	30°C, 48 h	99.5%–99.8%	Kim et al. (2005)
Rb1→Gypenoside LXXV	Fungus *E*. *vermicola* CNU 120806	pH 5.0, 50°C	95.4%	Hou et al. (2012)
Rb1→Ginsenoside XVII→Ginsenoside F2	*Intrasporangium* sp. GS603	27°C, 160 rpm, 72 h	—	Cheng et al. (2007)
Rb1→Ginsenoside F2	Rat Intestinal *Enterococcus gallinarum*	pH 7.0, 40°C	45%	Yan et al. (2021)
Rb1→Compound K	*L*. *mesenteroides* KFRI 690	37°C, 96 h	97.8%	Park et al. (2012)
Rb1→Compound K	Fungi *Arthrinium* sp. GE 17–18	30°C, 24 h	100%	Fu et al. (2016)
Rb1→3-keto and dehydrogenated C-K	*P*. *bainier* sp. 229	28°C, 5 days	—	Zhou et al. (2018)
Rb1→Rd→Rg3	*Microbacterium* sp. GS514	30°C, 48 h	41.4%	Cheng et al. (2008)
Rb1→Rd→Rg3	Bacterium *Burkholderia* sp. GE 17–7	pH 7.0, 30°C, 15 h	98%	Fu et al. (2017)
Rb1→Rd→Rg3	Bacterium *Flavobacterium* sp*.* GE 32	30°C, 72 h	—	Fu, (2019)
Rb1→Rd, Re→Rg2, Rg1→Rh1, Ginsenoside F1	*Cellulosimicrobium* sp. TH-20	pH 7.0, 30°C, 5 days	38%–96%	Yu et al. (2017)
Rb1→Compound K, Rg1→F1	*Cladosporium cladosporioides*	pH 7.0, 30°C	74.2%, 89.3%	Wu et al. (2012)
Rc→Rg3	*Leuconostoc* sp. BG78	37°C, 96 h	70%–75%	Ten et al. (2014a)
Rc→C-MC1	*Sphingopyxis* sp. BG97	37°C, 72 h	75%	Ten et al. (2014b)
Rd→Compound K	*Lactobacillus pentosus* DC101	pH 7.0, 30°C, 3 days	97%	Quan et al. (2010)
Rg1→25-OH-20(*S/R*)-Rh1	*Cordyceps Sinensis*	28°C, 150 rpm, 6 days	82.5%	Sui et al. (2020)
Saponins→mainly Rg3, F2, Compound K	Human fecal microflora	37°C, 24 h	—	Wan et al. (2013)
Rb1, Rb2, Rb3, Rc→Compound K	Human intestinal bacteria	37°C, 48 h	83.5%–88.7%	Zheng et al. (2021)
***In vivo*** **Transformation**				
Rb1→Rg3, Rh2→ Protopanaxadiol	Rat intestinal microbiota	Rat feces	—	Qian and Cai, (2010)
Rb1→Rb1+O	Rat plasma and urine	Plasma and urine	—	Wang et al. (2015a)
Rb1→Rd→Protopanaxadiol	Rat intestinal microbiota	Plasma, urine, and feces	—	Kang et al. (2016)
Rg1→Rg1+O	Rat plasma and urine	Plasma and urine	—	Wang et al. (2016)
Rg1, Re, Rf→Rh1→Protopanaxatriol	Rat intestinal microbiota	Plasma, urine, and feces	—	Dong et al. (2018)
Rg1, Re→Rh1, Rg2→F1, Rh1, Rg1	Human stomach and intestine	Plasma and urine	—	Tawab et al. (2003)
Rb1, Rc, Rd→Rg3, F2→Rh2, Compound K→Protopanaxadiol	Human intestinal microbiota	Plasma	—	Wan et al. (2016)

### Enzymatic transformation

**FIGURE 3:**
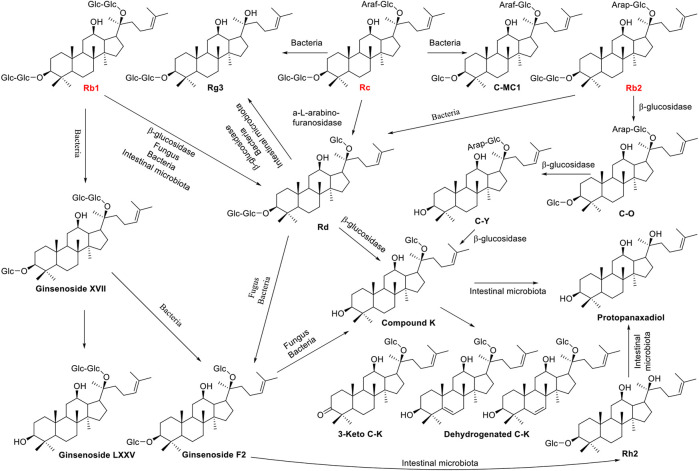
Schematic illustration of biotransformation of major PPD-type ginsenosides Rb1, Rb2, and Rc into minor ginsenosides.

Ginsenosides Rb1, Rb2, and Rc belong to protopanaxadiol (PPD) triterpenoid saponins, which are further modified by the glycosidation at the positions of C-3 and C-20 with different sugar moieties. The variable origin of microbial β-glucosidase determines the position and efficiency of deglycosylation. *Microbacterium esteraromaticum* derived β-glucosidase bgp1 catalyses ginsenoside Rb1 into 20(S)-Rg3 *via* intermediate product Rd, while β-glucosidase bgp3 transforms Rb1 into Compound K (C-K) *via* Rd (Quan et al., 2012b; Quan et al., 2012d). Intriguingly, crude glycosidase obtained from *Leuconostoc mesenteroides* DC102 transforms Rb1 into compound K with a yield of 99% after 3 days cultivation (Quan et al., 2011). In addition, an enzyme immobilization method was developed for the effective biotransformation of Rb1to Rd, and the catalytic efficiency of the immobilized β-glucosidase from *Aspergillus niger* was 3.30-fold higher than that of the free enzyme (Wu et al., 2021). Ginsenoside Rb2 can be transformed to Rd in the treatment of α-L-Arabinopyranosidase (Kim et al., 2020). While, after coculture Rb2 with β-glucosidase from *M. esteraromaticum* or *Armillaria mellea* mycelium, the product compound K was obtained via intermediate compounds Y, Rd, and C-O (Quan et al., 2012a; Kim et al., 2018). The a-L-arabinofuranosidase purified from *thermarum* DSM5069 catalyses ginsenoside Rc to Rd with a high yield of 99.4% (Xie et al., 2016). The biotransformation pathways were shown in Figure 3.

**FIGURE 4:**
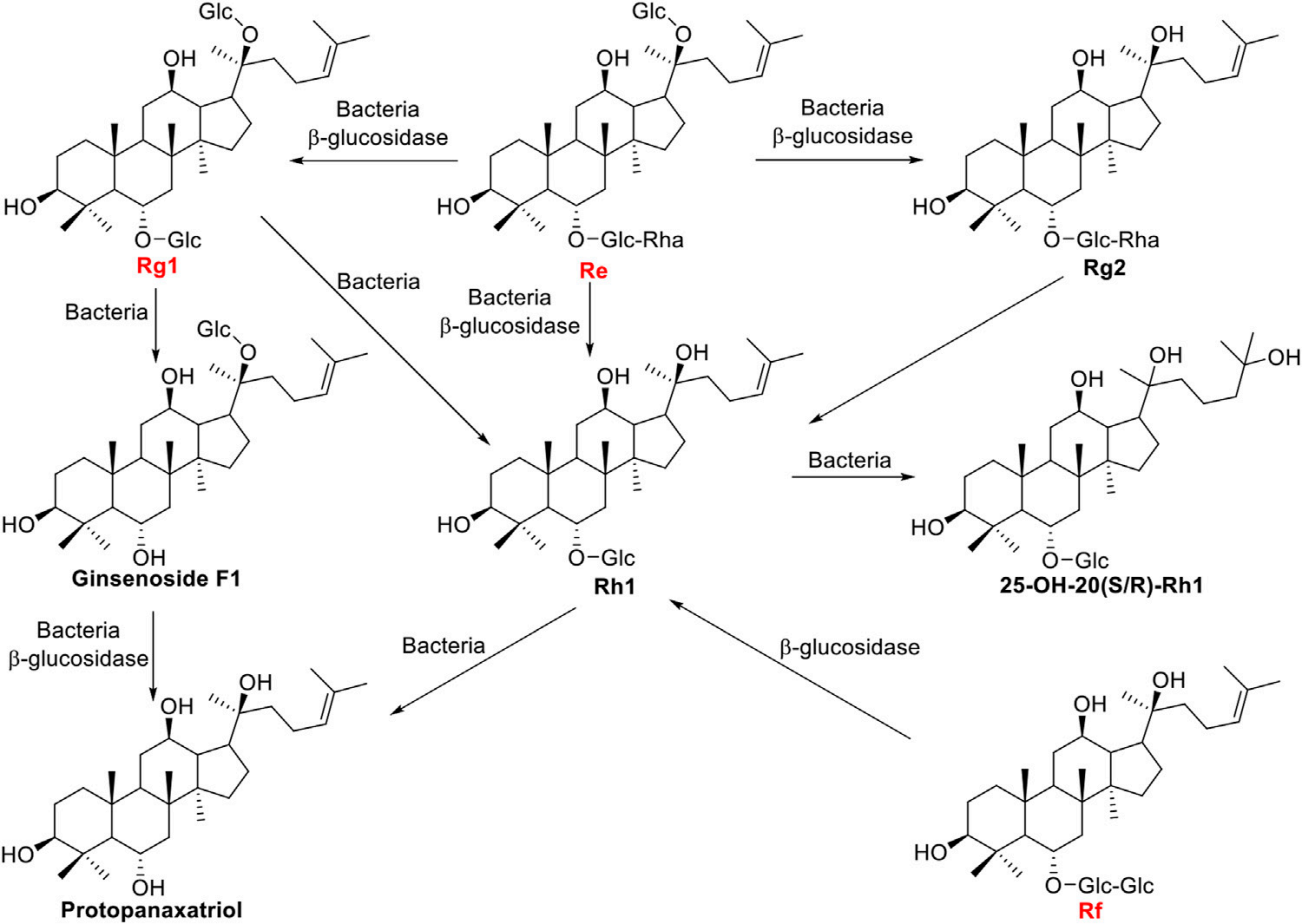
Schematic illustration of biotransformation of major PPT-type ginsenosides Re, Rf, and Rg1 into minor ginsenosides.

Ginsenosides Re, Rf, and Rg1 are another type of major ginsenosides belong to protopanaxatriol (PPT) triterpenoid saponins, and the positions of C-6 and C-20 are glycosidased with different sugar moieties. The β-glucosidase bgp1 gene consists of 2,496 bp encoding 831 amino acids which have homology to the glycosyl hydrolase families 3 protein domain. Recombinant β-glucosidase bgp1 transformed ginsenosides Re and Rg1 to ginsenosides Rg2 and Rh1, respectively (Quan et al., 2012c). A β-glucosidase gene isolated from *A*. *niger*, *bgl1*, was able to transform ginsenoside Rf into Rh1 (Ruan et al., 2009). The β-glucosidase finally transform Rh1 into PPT with a yield of 90.4% (Liu et al., 2010). Another β-glucosidase gene *bglSk*, isolated from *Sanguibacter keddieii*, consists of 1,857 bp and revealed significant homology to that of glycoside hydrolase family 3, which could convert major ginsenosides Rb1, Rb2, Rc, Rd, Re, and Rg1 into rare ginsenosides such as Compound Y, C-Mc, Compound K, Rg2(S), and F1. Kim et al. (2012) found *bglSk* could completely convert the Rg1 into F1. The biotransformation pathways were shown in Figure 4.

Collectively, the different β-glucosidase showed specialized catalysed position, and β-glucosidase bgp1 prefers to hydrolyse the glucosides at C-20 position, while β-glucosidase bglSk recognizes C-3 and C-6 position. However, β-glucosidase bgp3 and β-glucosidase isolated from *A*. *niger* do not show selectivity at C-6 and C-20.

### Microbial transformation

Microbial transformation is effective in modifying ginsenosides to obtain new chemical derivatives and is also a major production method of minor ginsenosides. The enzymatic transformation showed advantages of a short reaction time, superior environmental protection, and high product yield and purity. However, the separation and purification processes of enzymes are high-cost and complicated, and the reaction conditions are strictly controlled due to the susceptible enzyme activity. In contrast, microbial transformation is characterized by wide applications and low costs, but a dearth of high selectivity and a long conversion time. Thus, the combination of enzymatic and microbial transformation of ginsenosides could warrant the actual production process.

*Burkholderia pyrrocinia* GP16, *Bacillus megaterium* GP27, and *Sphingomonas echinoides* GP50 were screened from 70 strains of aerobic bacteria with β-glucosidase activity, and they almost completely transformed Rb1 to Rd (Kim et al., 2005). With the aid of bacteria *L*. *mesenteroides* KFRI 690 or Fungi *Arthrinium* sp. GE 17–18, ginsenoside Rb1 can be converted to Compound K efficiently with yields of 97.8% and 100%, respectively (Park et al., 2012; Fu et al., 2016). In addition, gypenoside LXXV and F2 were finally obtained *via* intermediate product Ginsenoside XVII without further conversion by Fungus *Esteya vermicola* CNU 120806 and bacteria *Intrasporangium* sp. GS603 transformation, respectively (Cheng et al., 2007; Hou et al., 2012). The scale-up fermentation was carried out using *Paecilomyces bainier* sp. 229, and ginsenoside Rb1 was converted to a known 3-keto C-K and two new dehydrogenated C-K metabolites (Figure 3), which were isolated through repeated silica gel column chromatography and high-pressure liquid chromatography (Zhou et al., 2018). Furthermore, several kinds of bacteria, such as *Microbacterium* sp. GS514, *Burkholderia* sp. GE 17–7, and *Flavobacterium* sp*.* GE 32, can transform Rb1 to Rg3 *via* the intermediate product Rd (Cheng et al., 2008; Fu et al., 2017; Fu, 2019). Rc was converted into minor ginsenosides Rg3 and C-MC1 with bacteria *Leuconostoc* sp. BG78 and *Sphingopyxis* sp. BG97, respectively (Ten et al., 2014a and Ten et al., 2014b). Sui et al. (2020) demonstrated that ginsenoside Rg1 could be thoroughly converted into 20(*S/R*)-Rh1 and 25-OH-20(*S/R*)-Rh1 by *Cordyceps Sinensis*, with a biocatalytic pathway established as Rg1→20(*S/R*)-Rh1→25-OH-20(*S/R*)-Rh1, and the molar bioconversion rate for total 25-OH-20(*S/R*)-Rh1 was 82.5%. Aside from bacteria and fungi, human fecal, and intestinal microflora could also transform ginsenosides. While human fecal microflora was prepared from a healthy Chinese man and subsequently incubated with *P. quinquefolius* saponins at 37°C for 24 h, three most abundant metabolites are identified with liquid chromatography/quadrupole time-of-flight mass spectrometry (LC–Q-TOF-MS) as 20(*S*)-ginsenoside Rg3, ginsenoside F2, and Compound K (Wan et al., 2013). Additionally, human intestinal bacteria were incubated with ginsenosides Rb_1_, Rb_2_, Rb_3_ and Rc at 37°C under anaerobic conditions, and ginsenoside Compound K was identified as the transformed product after 48 h with transformation rates of 83.5%, 88.7%, 85.6%, and 84.2%, respectively (Zheng et al., 2021).

### *In vivo* transformation

**FIGURE 5:**
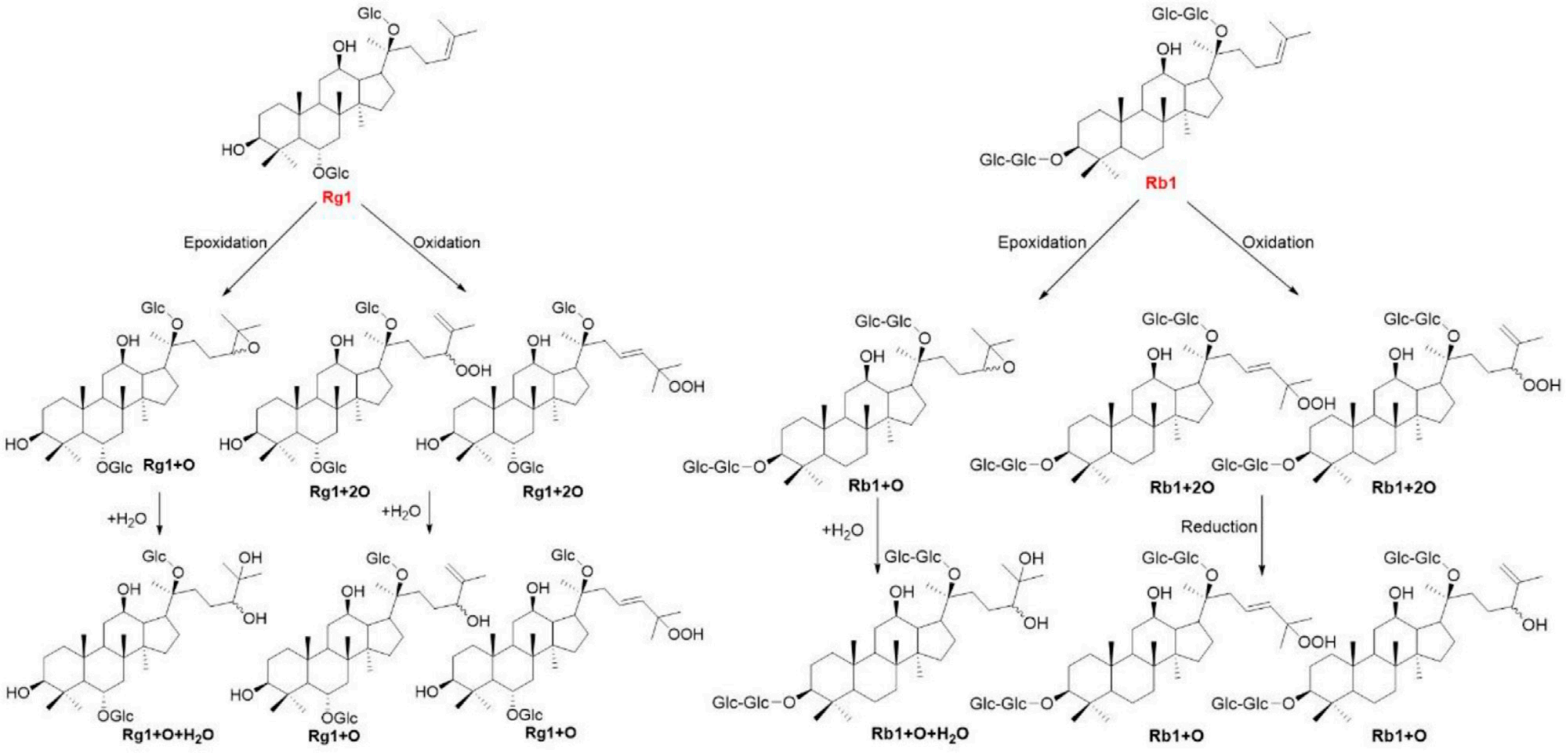
The oxygenated metabolites involved in *in vivo* biotransformation pathways of ginsenosides Rb1 and Rg1.

Gut microbiota mainly transform prototype ginsenosides into rare bioactive metabolites. Unlike *in vitro* enzyme and microbial transformation, the ginsenosides underlying anaerobically with pooled gut bacteria resulted in some novel metabolites in the plasma, bile, urine, and feces. After Rb1, Rg3, and Rh2 were administered to male Sprague Dawley rats at a dose of 100 mg/kg body weight, Rb1 and Rg3 could be metabolized to Rh2, while Rb1 could be metabolized to Rg3. The final products of Rb1, Rg3, and Rh2 were protopanaxadiol and monooxygenated protopanaxadiol (Qian and Cai, 2010). To further clarify the role of microbiota on metabolism of Rb1, ginsenoside Rb1 was administered to normal and antimicrobials treated rats, and the metabolites of Rb1, such as Rd, F2, and Compound K were detected in normal rat plasma but not in antimicrobials treated rats (Kang et al., 2016). Oxygenated metabolites have been considered as the major circulating metabolites of ginsenosides. After ginsenosides Rb1 and Rg1 were oral administered to rats for 24 h, totally 10 and 9 oxygenated metabolites were characterized by UHPLC-QTOF MS analysis, respectively (Figure 5) (Wang et al., 2015a; Wang et al., 2016). The degradation of ginsenosides has been thoroughly investigated in animals and *in vitro* using enzymes and microbiota, thus the elucidation of metabolites reaching the systemic circulation in human is of great importance. Six healthy male volunteers ingested 1 g of *P. quinquefolius* twice a day for 7 days. Totally, 5, 10, and 20 metabolites were detected in plasma, urine, and feces, respectively. And Compound K is found to be the major metabolite in all three samples (Wan et al., 2016).

## Pharmacological activities of ginsenosides from *P*. *quinquefolius*

### Anti-obesity and diabetes

PPD and PPT types of ginsenosides were purified from the leaves of *P. quinquefolius*, and the porcine pancreatic lipase activity was determined *in vitro*. PDG inhibited the pancreatic lipase activity in a dose-dependent manner at the concentrations of 0.25–1 mg/ml, while PPT showed no inhibitory activity. Moreover, PPD was effective in preventing and healing obesity, fatty liver and hypertriglyceridemia in mice fed with a high-fat diet (Liu et al., 2010b). Another clinical study indicated that the oral intake of *P. quinquefolius* extract with 1 g/meal (3 g/day) significantly reduced HbA1c and fasting blood glucose, and systolic blood pressure was also lowered (Vuksan et al., 2019). A dammarane from acid hydrolysates of *P. quinquefolius* total saponins, named 20(*R*)-dammarane-3β,12β,20,25-tetrahydroxy-3β-O-β-D-glucopyranoside, exhibited significantly inhibitory activity against α-glucosidase, and the IC_50_ value [(0.22 ± 0.21) μmol/L] was about 43-fold lower than the positive control acarbose, indicating the potential effects of saponins on diabetes (Han et al., 2020). After a 5-weeks treatment of malonyl ginsenosides from *P. quinquefolius*, the fasting blood glucose (FBG), triglyceride (TG), total cholesterol (TC), low-density lipoprotein cholesterol (LDL-C), nonesterified fatty acid (NEFA), alanine transaminase (ALT), and aspartate transaminase (AST) levels were significantly reduced and glucose tolerance and insulin resistance were improved (Liu et al., 2021). IRS1/PI3K/Akt and IRS1/PI3K/Akt pathways are involved in the anti-T2DM effects of malonyl ginsenosides.

### Anti-tumors

20(S)-PPD is a metabolite of ginseng saponin of *P. quinquefolius*, which significantly inhibited the growth and induced cell cycle arrest in HCT116 cells. An *in vivo* study showed that when i.p. administered (30 mg/kg) PPD once every 2 days for 3 weeks, xenograft tumor growth in athymic nude mice bearing HCT116 cells were inhibited (Gao et al., 2013). A structure-function relationship study indicated that sugar numbers within a ginsenoside exerted an inverse impact on tumor cells, and the sugar moiety at C-6 possess higher anti-cancer activity than that with linkages at C-3 or C-20, due to the increased steric hindrance to target proteins after C-6 was sugar substituted (Qi et al., 2010).

The number and position of hydroxyl groups in ginsenosides also affect their pharmacological activities. The substitution of hydroxyl or methoxyl groups at C-25 increases the anti-tumor effects of ginsenosides. Compared with 20(S)-Rh2, 20(S)-PPD and 20(S)-Rg3, 20(S)-25-OH-PPD showed the most apoptotic, antiproliferative, cell cycle arrest, and tumor growth inhibition effects *in vivo* (Wang et al., 2008b). In addition, usually 20(S) stereoisomers of ginsenosides show stronger chemopreventive effects than 20(R) stereoisomers (Qi et al., 2010).

### Anti-neurodegenerative diseases

When fifty-two healthy volunteers (40–60 years old, mean age 51.63) received 200 mg of *P. quinquefolius* or a matching placebo for 1, 3, and 6 h according to a double-blind, placebo-controlled, balanced, crossover design, the result showed that cognitive performance on “Working Memory” was significantly improved after treatment for 3 h (Ossoukhova et al., 2015). In addition, Cereboost™, an extract of *P. quinquefolius* extract, restored Aβ1-42 which insulted downregulation of brain microtubule-associated protein 2 and synaptophysin as well as acetylcholine concentration, thus recovered the cognitive function (Shin et al., 2016). When APP/PS1 AD mice was administered by pseudoginsenoside-F11 at 8 mg/kg for 4 weeks, the expressions of β-amyloid precursor protein (APP) and Aβ1-40 in the cortex and hippocampus were significantly inhibited, and the activities of superoxide dismutase (SOD) and glutathione peroxidase (GSH-Px) were restored (Wang et al., 2013a). Additionally, pseudoginsenoside-F11 exerts anti-Parkinson effects through inhibiting free radical formation and stimulating endogenous antioxidant release in a 6-hydroxydopamine-lesioned rat model (Wang et al., 2013b).

Experimental autoimmune encephalomyelitis (EAE) is a commonly used experimental model for the demyelinating disease, multiple sclerosis (MS). An aqueous extract of ginseng (150 mg/kg body mass) was oral administered to MOG (35–55) peptide induced EAE mice, and the clinical signs of EAE, TNF-α expression, and iNOS and demyelination scores were significantly improved compared with model mice (Bowie et al., 2012). Pseudoginsenoside-F11 (4 and 8 mg/kg bw twice at a 4 h interval) significantly mitigated anxiety-like behavior in methamphetamine-induced rats, shortened the time of immobility in forced swimming test, and significantly decreased the number of errors in the T-maze test (Wu et al., 2003).

### Others

The saponins from the leaves of *P. quinquefolius* showed a renoprotective effect in a mouse model of cisplatin-induced acute kidney injury. The further mechanism study clarified that saponins administration significantly suppressed the protein expression levels of Nox4, cleaved-Caspase-3, cleaved-Caspase-9, Bax, NF-κB, COX-2, and iNOS (Ma et al., 2017).

A MI/R model was constructed to investigate whether *P. quinquefolius* saponins decrease no-reflow phenomenon via suppression of inflammation, and the results showed that the inhibition of NLRP3 inflammasome *via* TLR4/MyD88/NF-κB signaling pathway is involved in *P. quinquefolius* saponins effects on cardiac functional improvement and pathological morphology changes of myocardium (Yu et al., 2021). Mice pretreated with saponins from the leaves of *P. quinquefolius* (150 or 300 mg/kg) by oral gavage for 7 days significantly reversed acetaminophen induced liver injury. Further study indicated that anti-oxidant, anti-apoptotic and anti-inflammatory activities were involved in its mechanism (Xu et al., 2017).

The heated *P. quinquefolius* could protect cell viability against H_2_O_2_-induced oxidative damage, and enhance the activities of superoxide dismutase and catalase dose dependently in V79-4 cells (Kim et al., 2007). Heat-processing reduced the content of ginsenosides Rb1, Re, Rc, and Rd, and increased the content of Rg2 and Rg3 in *P. quinquefolius*. After 2 h steaming, the percent content of ginsenoside Rg3 increased from 0.06% to 5.9%, and Rg3 showed the best antiproliferative effects in human breast cancer cell line MCF-7 *via* arresting cancer cells in G1-phase (Wang et al., 2008a).

**FIGURE 6:**
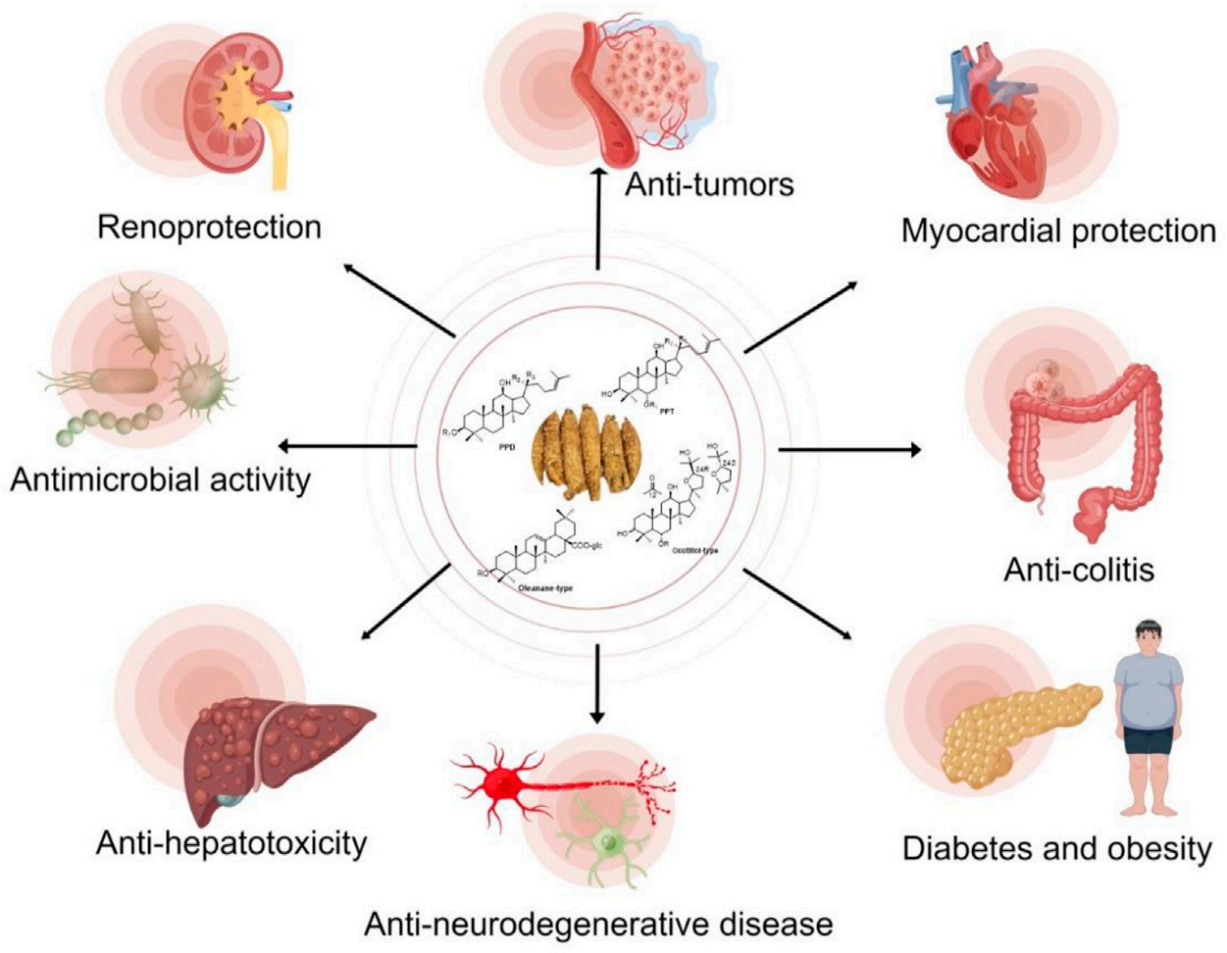
Biological and pharmacological activities of *P. quinquefolius* and its derived ginsenosides.

Ginsenoside C-Y can be used as a potential botanical agent to protect premature skin from UVB-induced photodamage and prevent skin hyperpigmentation (Liu et al., 2019). Taken together, *P. quinquefolius* and its derived ginsenosides possess a variety of pharmacological activities (Figure 6), which is a promising medicinal plant for human health.

## Conclusion and perspectives

Collectively, recent advances on the cultivation, chemical diversity, biotransformation, pharmacological, and clinical studies of *P. quinquefolius* were summarized in this review. A total of 75 naturally occurring ginsenosides have been identified from the roots, leaves and stems, flower buds, and fruits of wild or cultivated *P. quinquefolius*. With the aid of advanced chemical and analytical techniques and the characterization of novel compounds, the diversity of ginsenosides is constantly revealed.

Major ginsenosides, the main components in *P. quinquefolius*, are usually difficult to be absorbed and exhibit low bioavailability. However, minor ginsenosides with relatively high bioavailability and pharmacological activities can be obtained by biotransformation. Some of *P. quinquefolius* associated bacteria, fungus or their enzymes were purified, with highly selectivity to the substituted sugar moieties in C-3, C-6 and C-20. The *in vitro* and *in vivo* metabolic pathways of major ginsenosides are also discussed. Moreover, the pharmacological activities of *P. quinquefolius* or its derived ginsenosides, including anti-tumor, anti-diabetes and obesity, anti-colitis, anti-hepatotoxicity, anti-neurodegenerative disease, myocardial, and renoprotection were exhibited and summarized.

In conclusion, *P. quinquefolius* is a very promising medicinal plant for the treatment of diverse diseases, while the greater attention of the following issues should be focused in the future: 1) Due to the low yields of naturally occurring minor ginsenosides, most of the novel compounds are not screened for their biological activities, and total or semi-synthesis and directional biotransformation may be efficient ways. 2) Although the pharmacological effects of some ginsenosides were investigated, the direct targets and mechanism are rarely discovered, which need to be further elucidated.
